# Choriodecidual Group B Streptococcal Inoculation Induces Fetal Lung Injury without Intra-Amniotic Infection and Preterm Labor in *Macaca nemestrina*

**DOI:** 10.1371/journal.pone.0028972

**Published:** 2011-12-21

**Authors:** Kristina M. Adams Waldorf, Michael G. Gravett, Ryan M. McAdams, Louis J. Paolella, G. Michael Gough, David J. Carl, Aasthaa Bansal, H. Denny Liggitt, Raj P. Kapur, Frederick B. Reitz, Craig E. Rubens

**Affiliations:** 1 Department of Obstetrics & Gynecology, University of Washington, Seattle, Washington, United States of America; 2 Global Alliance to Prevent Prematurity & Stillbirth, Seattle, Washington, United States of America; 3 Department of Pediatrics, University of Washington, Seattle, Washington, United States of America; 4 Washington National Primate Research Center, Seattle, Washington, United States of America; 5 Ross University School of Medicine, Dominica, West Indies; 6 Department of Biostatistics, University of Washington, Seattle, Washington, United States of America; 7 Department of Comparative Medicine, University of Washington, Seattle, Washington, United States of America; 8 Department of Laboratories, Seattle Children's, Seattle, Washington, United States of America; 9 Center on Human Development and Disability, University of Washington, Seattle, Washington, United States of America; 10 Division of Infectious Disease, Seattle Children's, Seattle, Washington, United States of America; Charité, Campus Benjamin Franklin, Germany

## Abstract

**Background:**

Early events leading to intrauterine infection and fetal lung injury remain poorly defined, but may hold the key to preventing neonatal and adult chronic lung disease. Our objective was to establish a nonhuman primate model of an early stage of chorioamnionitis in order to determine the time course and mechanisms of fetal lung injury *in utero*.

**Methodology/Principal Findings:**

Ten chronically catheterized pregnant monkeys (*Macaca nemestrina*) at 118–125 days gestation (term = 172 days) received one of two treatments: 1) choriodecidual and intra-amniotic saline (n = 5), or 2) choriodecidual inoculation of Group B *Streptococcus* (GBS) 1×10^6^ colony forming units (n = 5). Cesarean section was performed regardless of labor 4 days after GBS or 7 days after saline infusion to collect fetal and placental tissues. Only two GBS animals developed early labor with no cervical change in the remaining animals. Despite uterine quiescence in most cases, blinded review found histopathological evidence of fetal lung injury in four GBS animals characterized by intra-alveolar neutrophils and interstitial thickening, which was absent in controls. Significant elevations of cytokines in amniotic fluid (TNF-α, IL-8, IL-1β, IL-6) and fetal plasma (IL-8) were detected in GBS animals and correlated with lung injury (p<0.05). Lung injury was not directly caused by GBS, because GBS was undetectable in amniotic fluid (∼10 samples tested/animal), maternal and fetal blood by culture and polymerase chain reaction. In only two cases was GBS cultured from the inoculation site in low numbers. Chorioamnionitis occurred in two GBS animals with lung injury, but two others with lung injury had normal placental histology.

**Conclusions/Significance:**

A transient choriodecidual infection can induce cytokine production, which is associated with fetal lung injury without overt infection of amniotic fluid, chorioamnionitis or preterm labor. Fetal lung injury may, thus, occur silently without symptoms and before the onset of the fetal systemic inflammatory response syndrome.

## Introduction

Preterm birth and the resulting neonatal morbidity and mortality represent a significant public health and economic burden to our society [1]. Intrauterine infection is present in most cases of the earliest preterm births and incites an inflammatory response believed to result in preterm labor and fetal lung injury [2]. Biological mechanisms initiating and propagating inflammation, preterm birth and fetal lung injury remain ill-defined and represent a major barrier to finding an effective treatment. The currently accepted paradigm is that bacteria ascend from the lower genital tract and sequentially colonize the choriodecidual space (external to the membranes and amniotic fluid) followed by trafficking into the amniotic fluid. Bacteria then induce cytokines, including interleukin-1β (IL-1β) and interleukin-8 (IL-8), which are thought to be a critical trigger of preterm labor and fetal lung injury predisposing the neonate to chronic lung disease [3], [4], [5].

Studying early events in microbial invasion of the amniotic cavity may help determine how a therapeutic might be best administered to successfully prevent preterm birth. An intramuscular or intravenous medication might be ideal to target choriodecidual bacteria and downregulate immune effectors. Recently, intra-amniotic infection has been suggested as an earlier step in the process, followed by secondary bacterial colonization of the fetal membranes (chorioamnion) resulting in chorioamnionitis [6]. If this scenario is more typical, then direct administration of a drug into the amniotic fluid via amniocentesis may be required. Both microbial and host factors, as well as inoculum size, are likely to play a role in whether bacterial colonization of the choriodecidual space results in intra-amniotic infection. In a pilot study in rhesus macaques, we have shown that Group B *Streptococcus* (GBS) inoculated into the choriodecidual space can traffic into the amniotic fluid, but required large and sometimes several inoculations [7]. The extent to which a small inoculum or transient infection might impact the mother or fetus' health is unknown, but may reflect the most common event leading to preterm birth in the setting of intrauterine inflammation [elevated amniotic interleukin-6 (IL-6)] and a negative amniotic fluid culture [8], [9], [10], [11].

Controversy also exists as to whether inflammation in the fetal membranes (chorioamnionitis) is associated with the most common form of neonatal chronic lung disease, bronchopulmonary dysplasia (BPD) [12], [13]. Although infection and inflammation can incite lung injury, the inflammatory response may also be protective by accelerating lung maturation and surfactant production. A recent meta-analysis of more than 15,000 subjects concluded that the pooled adjusted odds ratio for an association between BPD and chorioamnionitis was 1.58 (95% CI 1.1–2.2) [14]. Considerable study heterogeneity and publication bias, however, prevented the authors from concluding that chorioamnionitis was a definite risk factor for BPD. Understanding the relationship between inflammation and fetal lung injury has greater significance, because the prevalence of BPD is increasing, particularly in very immature infants who may have little or no evidence of respiratory distress syndrome. This variant has been called “new BPD” and is thought to be triggered by inflammation and intrauterine cytokines, which arrest alveolarization and inhibit growth of pulmonary vasculature [15], [16].

To investigate early factors involved in the initiation of intrauterine inflammation and fetal lung injury, we used a chronically catheterized pregnant nonhuman primate model (pigtail macaque; *Macaca nemestrina*). The nonhuman primate shares many important features with human pregnancy including uterine anatomy, singleton gestation, hemochorial placentation, and microbial communities within the vagina. We infused GBS, an organism known to cause preterm birth and neonatal invasive disease [17], [18], into the choriodecidual space via a catheter placed between the uterine muscle and membranes (external to amniotic fluid) overlying the lower uterine segment. We performed Cesarean section in the first week after choriodecidual inoculation to study early events associated with intrauterine infection.

## Results

### Uterine Activity

The mean gestational age of inoculation was 136 days (range: 131–139 days) and delivery was 141 days from conception (range: 134–145 days) corresponding to approximately 80% of a typical *M. nemestrina* pregnancy in our colony delivering at 172 days. Prior to GBS or saline inoculation, the uterus was quiescent in all animals. Peak uterine activity after inoculation was not significantly different between GBS and saline groups, but two GBS animals developed labor based on the finding of cervical change associated with more intense contractions (Table 1). In one case, a building contraction pattern (4,000–5,000 mmHg•sec/hr) and cervical change prompted Cesarean section three days after GBS infusion. In the second animal, slow cervical change occurred in the setting of episodic contractions. No cervical change or evidence of labor occurred in the remaining three GBS animals or any saline controls.

**Table 1:** Uterine Activity, Cytokines and Prostaglandins.

Measure	Post-inoculation Peak		p-value
	Saline (n = 5)	GBS (n = 5)	
Uterine activity (mmHg•sec/hr)	1,533.4 (880.6)	2,453.5 (2,362.5)†	0.70
	*Amniotic Fluid (ng/ml)*		
IL-1β	0.004 (0.006)	0.1 (0.2)	0.04*
TNF-α	0.02 (0.03)	0.6 (0.7)	0.01*
IL-6	9.9 (4.8)	72.6 (34.3)	0.003*
IL-8	1.3 (0.6)	13.0 (9.9)	0.001*
PGE_2_	0.6 (0.8)	0.7 (0.7)	0.71
PGF_2αα_	0.6 (0.4)	1.2 (1.3)	0.48
Total MMP activity (pmol/min)	25.9 (5.3)	29.1 (11.6)	0.59
	*Fetal Plasma (pg/ml)*		
IL-1β	0.0 (0.0)‡	0.0 (0.0)	-
TNF-α	0.7 (1.2)‡	0.1 (0.2)	-
IL-6	1.8 (0.8)‡	4.9 (4.5)	0.06
IL-8	309.5 (186.3)‡	1,569.2 (1,081.2)	0.03*

Table presents the mean (SD). Values for amniotic fluid analyses are ng/ml or pmol/min (MMP activity) and for fetal analyses are pg/ml. P-values are based on ANOVA models fit on log-transformed data except total MMP activity, which did not require log transformation.

*p<0.05.

† N = 4 for uterine activity in GBS group due to technical issues and insufficient data collection in one case.

‡ N = 3 for fetal plasma of saline controls.

### Cultures and GBS PCR

Cultures of the amniotic fluid and fetal membranes, lungs, blood and cerebrospinal fluid were performed to correlate findings with bacterial trafficking (Table 2). Culture of the chorioamnion at the inoculation site revealed low-level GBS [100–1,000 colony forming units/milliliter (CFU)] in two cases; one animal had no evidence of labor and the second had slow cervical change with episodic contractions. The inoculation site was culture negative in the other GBS animal in labor. Cultures of the amniotic fluid (∼10 samples tested/animal) and fetal tissues were negative in all animals, with the exception of suspected contaminants in a few cases (e.g. *Streptococcus viridans*). To determine whether low levels of GBS might be present in the amniotic fluid that was not detected by culture, quantitative real-time PCR was performed on serially sampled amniotic fluid at three points in the course of the experiment including the day prior to inoculation, day of Cesarean section and once during the course of the experiment. All amniotic fluid samples were negative with the lower limit of assay detection being 10 copies per PCR reaction.

**Table 2:** Amniotic Fluid and Fetal Cultures.

Group	AF	Membrane		Fetal Meninges	Fetal Lung	Fetal CSF	Fetal blood
		Inoculation site	Fundus				
Saline 1	None	None	None	None	None	None	None
Saline 2	None*	None	None	None	None	None	None
Saline 3	None	None	None	None	None	None	None
Saline 4	None*	None	None	None	None	None	None
Saline 5	None	None	None	None	None	None	None
GBS 1	None*	None	None	None	None	None	None
GBS 2	None	None	None	None	None	None	None
GBS 3	None	None	None	None	None	None	None
GBS 4	None	1,000 GBS, <100 *S. aureus*	None	None	None	None	None
GBS 5	None	100 GBS	None	None*	None*	None	None

Above values represent CFU/ml.

*A suspected contaminant (coagulase negative *Staphylococcus*, *S. viridians*) was noted in one AF culture of two saline controls and one GBS animal with all subsequent cultures being negative. Multiple suspected contaminants were noted in fetal tissue cultures from GBS #5 collected at the time of necropsy by an ill pathologist, who was coughing and wearing a mask (e.g. *Streptococcus gordonii*, *Neisseria* species).

AF, amniotic fluid; CSF, cerebrospinal fluid.

### Fetal Lung Injury

**Figure 1:**
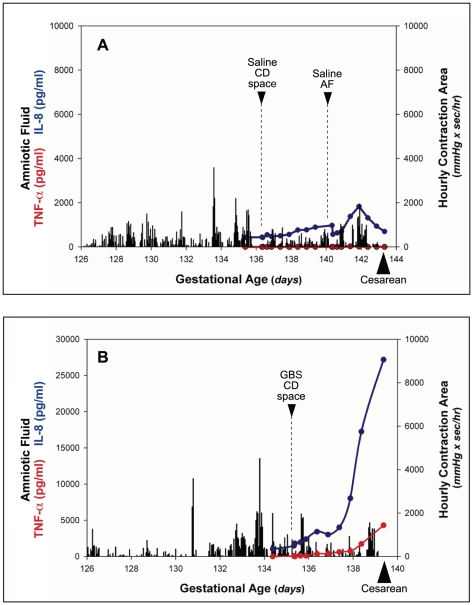
Histopathology of the fetal lungs. Hematoxylin and eosin stained histologic sections of fetal lung are shown for a saline control (A; lung score = 0) and GBS animal with severe fetal lung injury (B; lung score = 4).

A representative fetal lung section from a GBS and saline control animal is shown in Figure 1. Fetal lung histology was scored on a scale of 0 (normal) to 4 (severe injury) by a board certified veterinary pathologist (H.D.L.) blinded to group assignment as previously reported [19]. There was evidence of fetal lung injury in four of the five GBS animals (lung scores = 2, 3, 3, and 4), as well as one control (lung score = 2). Fetal lung injury varied in severity and distribution. Injury was defined as an aggregate of histologic changes including accumulation of inflammatory cells, evidence of necrosis, inflammatory related tissue thickening, collapse or other injury such as fibrin exudation or hemorrhage. The distribution of injury varied with severity but involved vascular and perivascular compartments as well as airway and alveolar compartments. In one control, fetal lung injury was an infarction of a lung tip and appeared histologically very different from controls and GBS lungs. In this case, hemorrhage was the predominant finding and thought to have occurred peri-mortem.

Inflammatory cells observed in the fetal lungs from GBS group included high numbers of neutrophils and macrophages, which is consistent with the pattern described in preterm infants at different stages of developing BPD [20]. In addition, there was increased staining density and thickened septa that was absent in saline controls. The most severe cases of fetal lung injury (fetal lung scores = 3, 3, and 4) correlated with the highest levels of amniotic fluid and fetal IL-8, but not other cytokines or prostaglandins tested (Table 1).

### Cytokines, Prostaglandins, and MMP

**Figure 2:**
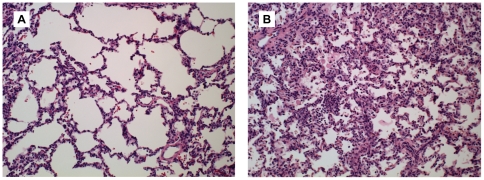
Uterine activity and amniotic fluid cytokines. Temporal relationships among inoculation of GBS or saline, uterine activity, and amniotic fluid (AF) cytokines (TNF-α, IL-8) are shown in a representative animal after saline inoculation (A) and in an animal after GBS inoculation (B), which developed moderate lung injury in the setting of minimal uterine activity. The x-axis represents gestational age in days ranging from the vascular implantation surgery until cesarean section. The y-axis is hourly contraction area (HCA; gray bars), or the level of amniotic fluid TNF-α (red line) or IL-8 (blue line). CD, choriodecidual; AF, amniotic fluid.

Temporal relationships between uterine activity, amniotic fluid cytokines and prostaglandins are depicted in one animal after GBS inoculation with moderate fetal lung injury and minimal uterine activity and a representative saline control (Figure 2). Saline infusion in either the choriodecidual space or the amniotic fluid of controls was not associated with an elevation in amniotic fluid cytokines. Following GBS choriodecidual infusion, amniotic fluid IL-1β, IL-8, IL-6 and TNF-α increased significantly compared to controls (Table 1; all p<0.05). GBS inoculation was not associated with a significant change in amniotic fluid prostaglandins or total matrix metalloproteinase activity during this four day experiment.

In two controls, fetal samples for cytokine analysis were not obtained due to either an inability to place the fetal catheter or clotting of the catheter. Fetal plasma IL-8 was significantly higher in GBS animals versus controls and correlated best with fetal lung injury of the fetal cytokines measured (p = 0.03). The GBS animal with the greatest degree of fetal lung injury (lung score = 4) also developed preterm labor and had a fetal IL-6 level of 11.3 pg/ml, which is diagnostic of the fetal systemic inflammatory response syndrome (FIRS) in humans [21]. In the other three GBS animals with fetal lung injury, the fetal IL-6 level (2.6, 3.1, 7.5 pg/ml) was below the threshold for FIRS. Fetal plasma IL-1β was undetectable in all but one animal and fetal plasma TNF-α was undetectable in all but two animals.

### Placental Histopathology

**Figure 3:**
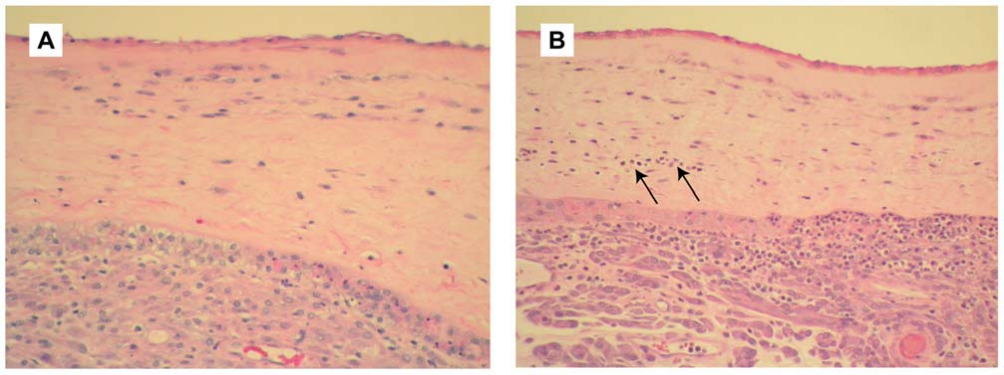
Histopathology of chorioamnion. Hematoxylin and eosin stained histologic sections of chorioamnion (fetal membranes) are shown for a saline control (A) and GBS animal with chorioamnionitis (B). Neutrophils in the chorion are indicated with arrows in panel B, as well as being abundant in the decidua.

Histopathology of the chorioamnion was reviewed by a pathologist (R.P.K.) blinded to group assignment. Representative tissues are shown in Figure 3 and results correlated with fetal lung score and labor in Table 3. Chorioamnionitis was diagnosed in two GBS animals with evidence of fetal lung injury (lung scores = 4 and 2); only one of these animals was in labor. Funisitis (inflammation of the umbilical cord) also occurred in one case, which correlated with the lung score of four. As previously noted, GBS was not detected in the amniotic fluid by either culture or PCR. Normal placental histology was noted in all controls and the remaining three GBS animals, two of which had signs of moderate fetal lung injury (lung score = 3 in both cases). In one GBS animal with fetal lung injury and a final diagnosis of normal histology, there were very rare scattered degenerating neutrophils within the membranes; this finding was subtle and not sufficient for making the diagnosis of chorioamnionitis.

**Table 3:** Fetal Lung Score, Cytokines and Chorioamnionitis in Each Animal.

Group	Fetal Lung Score	Peak AF		Peak Fetal Plasma		Inoculation Site Culture	Chorio-amnionitis	Labor
		IL-6 (ng/ml)	IL-8 (ng/ml)	IL-6 (pg/ml)	IL-8 (pg/ml)			
Saline 1	0	8.3	1.0	*	*	No growth	NO	NO
Saline 2	2	16.0	1.6	2.0	523.4	No growth	NO	NO
Saline 3	0	3.0	0.5	*	*	No growth	NO	NO
Saline 4	0	10.3	1.6	0.9	182.3	No growth	NO	NO
Saline 5	0	11.7	1.9	2.4	223.0	No growth	NO	NO
GBS 1	0	13.8	3.6	0	3,142.8	No growth	NO	NO
GBS 2	2	102.6	6.3	7.5	558.1	No growth	YES	YES
GBS 3	3	80.8	8.5	2.6	1,606.5	No growth	NO	NO
GBS 4	3	88.1	27.2	3.1	1,975.2	1,000 GBS	NO	NO
GBS 5	4	77.9	19.3	11.3	563.5	100 GBS	YES	YES

*In these two animals, fetal samples were not obtained.

## Discussion

Our study objective was to determine early biological events in choriodecidual infection and temporal relationships between bacterial trafficking, chorioamnionitis and fetal lung injury. We, therefore, performed Cesarean section a few days after initial inoculation and in the absence of labor in all but two cases. Despite relative uterine quiescence and minimal numbers of GBS organisms cultured from membranes at the inoculation site, significant fetal lung injury was detected in four of five animals just four days after choriodecidual inoculation. The lung injury could not be attributed to live bacteria, because amniotic fluid cultures and PCR were negative in all animals. This suggests that fetal lung injury may occur silently, predisposing the neonate to bronchopulmonary dysplasia, without presenting any warning signs to the obstetrician until the relatively late development of preterm labor.

**Figure 4:**
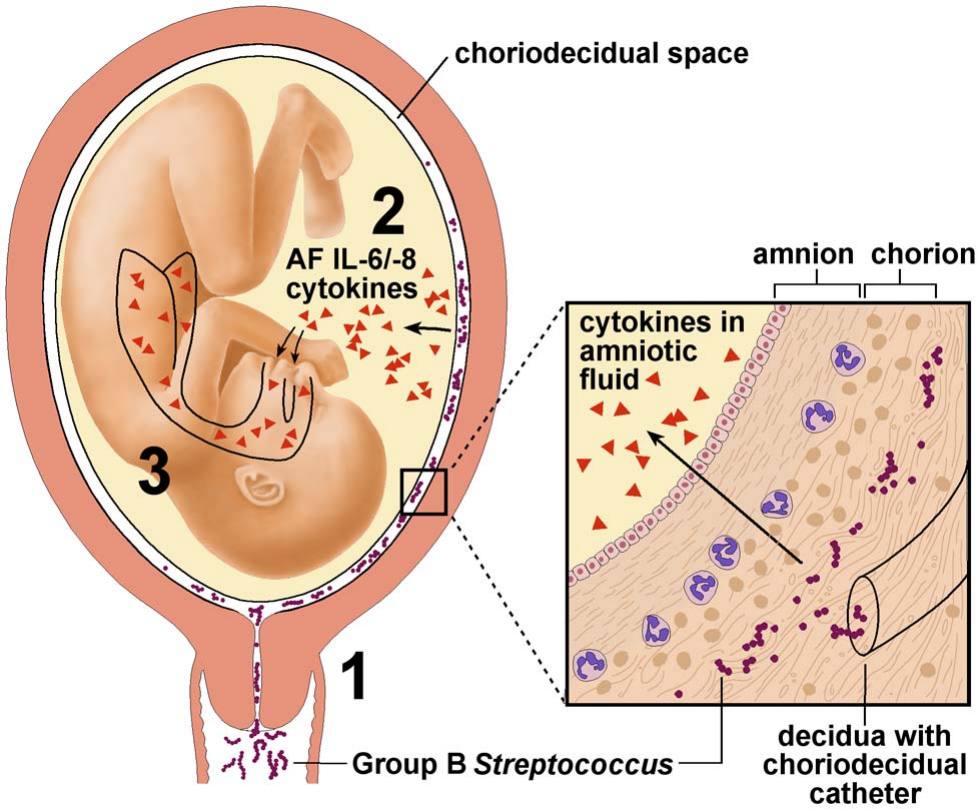
Our conceptual model. 1. Bacteria from the lower genital tract ascends into the choriodecidual space. 2. Inflammatory mediators (e.g. IL-8) produced by decidua and/or membranes diffuse into amniotic fluid and the fetal lung. 3. Fetal lung injury is induced by inflammatory mediators. IL, Interleukin; AF, amniotic fluid.

Elevation of amniotic fluid cytokines in GBS animals of our study is consistent with the hypothesis of others that aspiration of inflammatory mediators from the amniotic fluid is a key factor contributing to the fetal lung injury leading to BPD, the leading cause of chronic lung disease in infancy in the United States [5], [16], [22], [23], [24]. Our study extends this work by revealing that fetal lung injury may occur without clinical signs or symptoms of preterm labor and no microbiologic or pathologic evidence of infection. This suggests that a placental or membrane immune response may clear a choriodecidual infection to allow the pregnancy to continue, but results in production of inflammatory mediators that induce fetal lung injury in a paracrine response (Figure 4). This may occur through diffusion of inflammatory mediators into the amniotic fluid and direct contact with fetal lungs or via diffusion into fetal blood at the maternal-fetal interface [25], [26]. The mechanisms involved in the pathway linking antenatal inflammation and chorioamnionitis to fetal lung injury remain unclear.

How fetal lung injury is initially mediated has not been explored previously, because amniotic fluid and cord blood samples are generally collected at the time of preterm labor and delivery, which our study suggests may be quite late in the process. The relatively low levels of fetal plasma cytokines in most GBS animals with fetal lung injury suggest that in the early stages, fetal lung injury may be more likely to occur through direct contact of the fetal lung with inflammatory mediators in the amniotic fluid. In one animal receiving two GBS inocula, an elevated fetal plasma IL-6 (11.3 pg/ml) meeting criteria for FIRS occurred; this level of fetal IL-6 has been reported to better predict BPD than inflammatory mediators in the amniotic fluid [27], [28]. However, marked fetal lung injury was noted in three other GBS animals despite fetal IL-6 levels that were fairly low in two cases (2–3 pg/ml). In contrast, amniotic fluid peak IL-6 levels were relatively high in all animals with lung injury (range: 77.9–102.6 ng/ml). Whether an elevated amniotic fluid IL-6 or another inflammatory mediator initiates fetal lung injury is unknown, but this pattern of inflammation may be a biomarker for the early stages of fetal lung injury; interestingly, it also models the clinical condition in up to 25% of preterm labor cases with an elevated amniotic fluid IL-6 and a negative culture and/or PCR result [8], [9], [10], [11]. This suggests that *in utero* fetal lung injury of some degree may be much more common than previously recognized.

The role of IL-8 as a biomarker or initiator of BPD is also unclear. IL-8 is a plausible candidate as a key trigger of injury, because it is a chemoattractant for neutrophils, which were the predominant leukocyte in affected fetal lungs. In our study, amniotic fluid and fetal plasma IL-8 was significantly elevated in the GBS group compared to controls. Two of the four GBS animals had an amniotic fluid IL-8 greater than 10.7 ng/ml, which is a threshold previously associated with BPD (range: 6.3–27.2 ng/ml) [24]. In a prior study in rhesus macaques, we have shown that daily infusions of a single cytokine including IL-8 and others (IL-6, IL-1β, TNF-α) were associated with neutrophil infiltrates in the fetal lungs; IL-8 and IL-6 infusions were also associated with interstitial lymphocytic aggregates, but this may have been a function of a longer exposure duration [29]. In the preterm fetal sheep model, infusion of recombinant sheep IL-8 induced a fivefold increase in monocytes and neutrophils, but did not induce lung maturation associated with arrest of lung development and BPD [30]. Blockade of lipopolysaccharide-induced IL-8 signaling (CXCR2 blocker) was also not effective in decreasing leukocytes in the bronchoalveolar lavage or cytokines in the fetal lung. These studies in sheep suggest that IL-8 may be more accurately described as a biomarker.

Matrix metalloproteinases have been linked to bronchopulmonary dysplasia and degradation of the fetal membrane extracellular matrix [31], [32]. Increased activity of matrix metalloproteinase-9 (MMP-9) likely plays a role in fetal lung injury, because elevated cord blood MMP-9 has been correlated with bronchopulmonary dysplasia severity and oxygen supplementation [33]. We found no changes in total matrix metalloproteinase activity in the amniotic fluid, which is consistent with both *ex vivo* and *in vivo* data suggesting that increased matrix metalloproteinase activity is localized within the membranes and fetus [34], [35]. In a pilot study of GBS choriodecidual infection in rhesus macaques, active MMP-9 only became elevated in the amniotic fluid of a single animal after a high dose GBS inoculum that resulted in preterm labor [36]. In early choriodecidual infection, local production and conversion to active enzyme within the fetal lungs may be a more likely mechanism of injury than direct exposure of the fetal lungs to matrix metalloproteinases in amniotic fluid.

The absence of chorioamnionitis in two cases with fetal lung injury sheds light on an important controversy surrounding whether chorioamnionitis is a risk factor for BPD [14], [23], [37], [38], [39]. Although early observational studies suggested an association, a subsequent cohort found no association [14]. Bronchopulmonary dysplasia began to be viewed as requiring “multiple hits”, which included an antenatal inflammation component, as well as postnatal factors like oxidative stress, ventilator injury, and proteases [38]. Likewise, studies have reported chorioamnionitis to be associated with both increased production and depletion of surfactant [40]. These conflicting reports suggest that the degree and duration of inflammation may be important in determining the outcome on surfactant production and possibly the outcome of fetal lung injury. Our results suggest that a normal histologic appearance of the fetal membranes does not preclude the possibility of a transient choriodecidual infection that may have triggered fetal lung injury. As we ended these experiments after four days, it is unclear whether the fetal lung injury observed would progress or resolve with further time *in utero* [41], [42]. Only survival studies with measurements of lung function after birth would address the question of whether this lung injury develops into a condition similar to human bronchopulmonary dysplasia with accelerated lung maturation, disordered structural development and impaired function.

Animal models have previously been used to model lung injury often by inducing end-stage disease. In the preterm fetal sheep model, the time course and major inflammatory effectors of endotoxin-induced fetal lung injury have been defined [41], [43], [44]. In contrast, this study focused on early events in lung injury, which may have significant relevance in investigation directed towards prevention. A major strength of this work is the use of an animal model with many similarities to both human pregnancy and fetal lung development. The nonhuman primate has a singleton fetus with a long gestational period (160–170 days), hemochorial placentation, and maternal-fetal inflammatory responses induced by infection and parturition, which mimic human pregnancy. Fetal lung injury in our model also occurs in the saccular stage of fetal lung development, which replicates the timing of human fetal lung injury leading to neonatal chronic lung disease. Other animal models (sheep, mice) are in a more advanced stage of lung development (alveolar) at the time of experimental injury, which may limit translation of results to human disease. Pulmonary morphologic and immune features in our model also emulate that in humans, but differ from many other species [45]. Both humans and nonhuman primates lack pulmonary intravascular macrophages present in the lungs of many species, making their lungs less susceptible to injury than other species (e.g. sheep, cattle, pigs).

One important study limitation is that microbial invasion of the amniotic cavity is likely a chronic process and the time course of infection depends on the organism and inoculum. Our results are more likely to reflect events associated with an immune response to a small or limited choriodecidual infection. A second limitation is that we terminated our experiments at 4 days; thus, we may have truncated a process leading to further fetal lung injury or maturation as the repair process begins. Third, the small sample size for fetal cytokine data in the saline group precluded confirming a possible association between fetal IL-8 and lung injury as suggested by our data and prior reports [5], [23], [46].

Mechanisms initiating and propagating fetal lung injury *in utero* are difficult to study after birth due to the many confounding clinical variables in the intensive care of preterm infants, such as the use of mechanical ventilation. Further studies at different time points may elucidate the natural course of fetal lung inflammation induced by choriodecidual bacteria and provide insight into how this response potentially contributes to neonatal lung injury. Additional soluble effectors associated with this process remain to be defined and could include previously implicated biomarkers of fetal lung injury including many chemokines (e.g. MCP-1, MIP-1β) [47], elastase [48], leukotriene B_4_ [48], fibronectin [49], MMP-8 [12], MMP-9 [50], [51], and complement C5a anaphylatoxin [52]. Overall, our model provides a unique opportunity to study the fetal origins of neonatal chronic lung disease induced by amniotic fluid inflammatory mediators, which can be triggered by many types of bacteria.

## Materials and Methods

### Ethics Statement

This study was carried out in strict accordance with the recommendations in the Guide for the Care and Use of Laboratory Animals of the National Research Council and the Weatherall report, “The use of non-human primates in research”. The protocol was approved by the Institutional Animal Care Use Committee of the University of Washington (Permit Number: 4165-01). All surgery was performed under general anesthesia and all efforts were made to minimize suffering.

### Animals and Study Groups

Ten chronically catheterized pregnant monkeys (*Macaca nemestrina*) at 118–125 days gestation (term = 172 days) received one of two experimental treatments: 1) choriodecidual and intra-amniotic saline infusions (n = 5), or 2) GBS choriodecidual inoculation (n = 5). Saline infusions were performed separately in the choriodecidual and amniotic fluid to confirm that saline would not stimulate cytokine production in either compartment. In two saline controls, fetal samples were not collected due to either an inability to place the fetal catheter during initial surgery or clotting of the fetal catheter. This resulted in three fetal cytokine analyses in the saline group. In one GBS case, technical problems led to only intermittent data collection and so the remaining uterine activity data was excluded for this animal.

In our model, pregnant pigtail macaques were time-mated and fetal age determined using early ultrasound. The tethered chronic catheter preparation was used for all *in vivo* experiments and is a major breakthrough in studying maternal-fetal immunologic responses [53], [54]. The animal was first conditioned to a nylon jacket/tether system for several weeks before surgery, which allowed free movement within the cage, but protected the catheters. On day 118–125 of pregnancy (term = 172 days) catheters were implanted into the maternal femoral artery and vein, fetal internal jugular vein, amniotic cavity, and choriodecidual interface in the lower uterine segment (between uterine muscle and fetal membranes, external to amniotic fluid). Fetal ECG electrodes and a maternal temperature probe were also implanted. After surgery, the catheters/electrodes were tracked through the tether system and cefazolin and terbutaline sulfate were administered to reduce postoperative infection risk and uterine activity. Both cefazolin and terbutaline were stopped at least 72 hours before experimental start (∼5 half-lives for terbutaline, 40 half-lives for cefazolin, >97% of both drugs eliminated). Cefazolin 1 gram was administered intravenously each day in saline controls to minimize chances of a catheter-related infection. Experiments began approximately two weeks after catheterization surgery to allow recovery (∼30–31 weeks human gestation). At our center, term gestation in the non-instrumented pigtail macaque population averages 172 days.

### Uterine Activity, Labor and Delivery

Intraamniotic pressure, fetal heart rate and maternal temperature were continuously recorded and digitized with a Powerlab System (AD Instruments, Colorado Springs, CO) connected to an Intel computer. Amniotic fluid pressure signals were processed after delivery using custom software to eliminate noise due to respiration or position changes. The area under each contraction (mmHg•sec/hr) was summed for each hour allowing calculation of the hourly contraction area, which is a measure of uterine activity. Labor was defined as progressive uterine activity associated with cervical effacement and dilatation. In the GBS group, Cesarean section was performed when uterine activity exceeded a 12,000 hourly contraction area for more than two hours and/or cervical change indicated labor. Cervical exams were performed on all animals the day prior to inoculation and then daily in the GBS group or every other day in the saline group until Cesarean section. Cesarean section was performed in all animals in order to optimize the collection of intact gestational tissues. In order to study early events following a choriodecidual infection, a Cesarean section was planned 4 days after the GBS inoculation. In saline controls the Cesarean section was performed 7 days after initial inoculation; saline was inoculated twice (day 0 and day 4) in alternating order into the choriodecidual space and amniotic fluid. This protocol was chosen to determine if saline inoculation induced production of inflammatory mediators when inoculated into either the amniotic fluid or choriodecidual space; no significant elevation was detected. In one GBS animal with no cervical change during the experiment, technical difficulties resulted in insufficient collection of data for analysis of uterine activity.

### Pathology and Lung Injury

After cesarean section, fetuses were euthanized by barbiturate overdose followed by exsanguination and fetal necropsy. Complete gross and histopathologic examination was performed on infants and placentas. The placenta was examined by a board-certified pediatric pathologist (R.P.K.) and fetal lungs examined by a board-certified veterinary pathologist (H.D.L.) with the each pathologist blinded to group assignment. Lung histologic sections were evaluated and scored, as previously described, using a semi-quantitative scale [19]. Components were scored on a scale of 0–4 (0 = normal) for inflammatory cells, necrosis, and inflammation including tissue thickening, collapse or other injury (e.g. fibrin exudation). Lung compartments scored were (1) vascular/perivascular; (2) bronchial/peribronchial; (3) alveolar wall; and (4) trichrome stain intensity positivity. Mononuclear inflammatory cells and neutrophils (Leder stain) within alveolar spaces were counted (5 random 40× fields). An overall severity score was generated.

### GBS and Bacterial Cultures

The microbiologist preparing the GBS and performing the bacterial cultures participates in a laboratory quality assurance program through the College of American Pathologists (CAP) and is licensed through the American Society of Clinical Pathologists (ASCP) and the American Society of Microbiologists (ASM). We inoculated a clinical isolate of GBS (Type III, COH-1) using 1×10^6^ of freshly grown mid-log phase bacteria inoculated into the choriodecidual space of the lower uterine segment. The COH-1 isolate was recovered from a neonate with meningitis and is highly virulent; it is the same strain used in prior experiments of intra-amniotic infection and pilot studies of choriodecidual GBS infection [7], [53]. Single colonies of minimally-passaged GBS from blood agar plates were transferred to sterile Todd Hewitt Broth and grown overnight. The GBS inoculum was prepared to a concentration of 10^6^ CFU/mL using the McFarland standard and a spectrophotometer. Bacterial concentration and purity was confirmed by colony counts on sheep blood agar with an average inoculum of 4.6×10^6^ CFU (range: 3.5–6.3×10^6^). In one GBS animal, the inoculum was inadvertently administered simultaneously with cefazolin. This GBS strain (COH-1) is rapidly killed by cefazolin, therefore the cefazolin was stopped. Three days later a second inoculum of GBS was given to the same animal.

Amniotic fluid was collected daily into anaerobic culture vials and equal volumes were sterilely inoculated in chopped-meat anaerobic broth as well as streaked for isolation on Brucella H/K and Chocolate agar plates. An additional volume was added to a glass microscope slide and allowed to air dry for Gram staining. Cultures grown anaerobically were placed within anaerobic culture jars and the anoxic environment was confirmed by aerobic indicators. Anaerobic plates were read after 5–7 days and chocolate plates grown under aerobic conditions were read after 72 hours. Any growth was subcultured and characterized according to established microbiological techniques.

Additional cultures were collected at the end of the experiment during Cesarean section and fetal necropsy. The maternal membrane fundus and inoculation site in the choriodecidual space were swabbed and placed in anaerobic culture tubes. Cerebral spinal fluid was aspirated in a sterile fashion and collected in anaerobic culture vials. Fetal lungs and meninges were sterilely swabbed and placed within anaerobic culture tubes. Fetal CSF and blood cultures were cultured as conducted with amniotic fluid. The above swab cultures were streaked for isolation on Brucella H/K and chocolate agar plates, and then placed in chopped meat broth for culture and grown according the procedure described above.

### Quantitation of Amniotic Fluid Cytokines, Prostaglandins, and Matrix Metalloproteinases

Amniotic fluid and maternal and fetal blood were sampled frequently before (−24 and −1 hour) and after GBS inoculation (+1, +6, +12, +24 hours, and then daily until delivery). Amniotic fluid and blood samples were centrifuged for 5 minutes at 1200 RPM and the supernatant frozen and stored at −80°C. Prior to freezing, ethylene diamine tetraacetic acid (EDTA) (8.7 mM) and indomethacin (0.3 mM) was added to samples saved for cytokine and prostaglandin quantitation, respectively. Cytokine (IL-1β, IL-6, IL-8, and TNF-α) levels were determined by Luminex multiplex technology using commercially available non-human primate cytokine kits (Millipore, Billerica, MA). Quantities of PGE_2_, and PGF_2α_ were determined using commercially available human ELISA EIA kits (Cayman Chemical, Ann Arbor, MI). Values were converted to pg/ml by comparison to a standard curve.

Analysis of matrix metalloproteinase activity was performed on amniotic fluid sampled and stored as described above using P126 (BioMol, Plymouth Meeting, PA), a MMP fluorogenic substrate [Mca-Pro-Leu-Gly-Leu-Dpa-Ala-Arg-NH_2_·AcOH, Mca = (7-methoxycoumarin-4-yl)acetyl; Dpa = N-3-(2,4-dinitrophenyl)-L-α,β-diaminopropionyl]. Briefly, samples were diluted in activity buffer (500 mM HEPES, 100 mM CaCl_2_, 0.05% w/v Brij-35, pH 7), pre-warmed to 37°C, and added to the fluorogenic substrate (10 µM). Quantification of the activity (pmol/min) was achieved using a standard curve generated with the cleaved fluorogenic product, P127 (BioMol, Plymouth Meeting, PA). Plate fluorescence (Ex.: 328 nm, Em.: 393 nm) was measured serially for four hours at 37°C and a standard curve generated of the cleaved fluorogenic product (P127; pmol/min). The units of total MMP activity are reported as pmol/min of cleaved product. Positive controls were run for each plate with either human recombinant MMP-9 or lysed fibroblasts.

### Quantitative Real-Time PCR

All primers were designed using Primer 3 software (Whitehead Institute for Biomedical Research, NJ). A standard curve was prepared by amplifying *CPSH_III_* sequence specific to the GBS strain, COH1. First, genomic DNA was purified from 500 µL of overnight GBS culture in TSB using the Gentra Puregene Yeast/Bact. Kit (Qiagen, Valencia, CA). Primers, 5′-CTT TGG AAG AGT GAG TTA G-3′ and 5′-TGA CCA ATT AGT GTA GCA TA-3′, were used to generate a 389-bp product from the *CPSH_III_* sequence [55]. The PCR amplification was performed in 25 µL reactions using MyTaq (Bioline, Taunton, MA, USA). The PCR conditions were as follows: initial DNA denaturation at 95°C for 60 sec followed by 35 cycles of 95°C for 30 s, 52°C for 30 sec, and 72°C for 40 sec. Primer specificity and quality was confirmed by gel electrophoresis of 5 µL PCR product on 1.5% agarose gel, stained with 0.02 µg/mL ethidium bromide. The PCR product was purified using MinElute Reaction Cleanup Kit (Qiagen, Valencia, CA) and the concentration was determined by Nanodrop. Amplicon concentration (amp/µL) was determined using the molecular weight of amplified product calculated with sequence manipulation suite and the DNA concentration [56].

For quantitative real-time PCR, 500 µL of amniotic fluid was centrifuged at 15,000×g for 5 minutes. Supernatant was removed and the pellet resuspended in 40 µL of cell lysis buffer (20% sucrose, 10 mM MgCl, 20 mM Tris pH 7.0, 0.05% Triton X-100, 0.5 units/µL mutanolysin). Cells were lysed at 37°C for 2–3 hours. Quantitative real-time PCR was conducted in 25 µL reactions consisting of 5 µL of above cell lysate, respective standard, or PCR-grade water (Ambion, Austin, TX); 0.25 µM primers: 5′-CAG TTG TAA GGA ATG TGG TAA AGG-3′ and 5′-AAA GTT GGC TTC AGC ATA GG-3′; and 1× SensiMix SYBR and Fluoroscein qRT-PCR Kit Mix (Bioline, Taunton, MA). A 500 seq/µL standard and 1,000 CFU/µL were used as positive controls for unknowns. The reaction conditions were as follows: DNA was denatured at 95°C for 10 minutes followed by 40 cycles at 95°C for 15 seconds, 55°C for 15 seconds, and 72°C for 15 seconds. Specificity of primers was confirmed with melting curve of product and gel electrophoresis with 10 µL of reaction product on a 2% agarose gel.

### Statistical Analysis

Study outcomes were quantities of uterine activity (mean 24-hour HCA), cytokines in amniotic fluid and fetal plasma (IL-1β, TNF-α, IL-6, IL-8), and amniotic fluid prostaglandins (PGE_2_, PGF_2α_) and matrix metalloproteinases (total MMP activity). Data was transformed by natural logarithm prior to analysis with the exception of matrix metalloproteinase data. We used one-way analysis of variance (ANOVA) to assess differences between the control and GBS groups with respect to each outcome. IL-1β and TNF-α in fetal plasma could not be assessed due to a large number of 0 values. All statistical analyses were conducted using Intercooled STATA 8.2 for Windows 2000 (StatCorp, College Station, TX). Significance was considered at p<0.05.
