# Correction: Choriodecidual Group B Streptococcal Inoculation Induces Fetal Lung Injury without Intra-Amniotic Infection and Preterm Labor in *Macaca nemestrina*

**DOI:** 10.1371/annotation/5abb9d73-7eca-4804-aad6-ebac580a926b

**Published:** 2012-08-13

**Authors:** Kristina M. Adams Waldorf, Michael G. Gravett, Ryan M. McAdams, Louis J. Paolella, G. Michael Gough, David J. Carl, Aasthaa Bansal, H. Denny Liggitt, Raj P. Kapur, Frederick B. Reitz, Craig E. Rubens

The images for Figures 1 and 2 were incorrectly switched. The image that appears as Figure 1 should be Figure 2, and the image that appears as Figure 2 should be Figure 1. The figure legends appear in the correct order. 

